# Differences in peripheral blood mononuclear cell gene expression and triglyceride composition in lipoprotein subclasses in plasma triglyceride responders and non-responders to omega-3 supplementation

**DOI:** 10.1186/s12263-019-0633-y

**Published:** 2019-04-25

**Authors:** Amanda Rundblad, Sunniva V. Larsen, Mari C. Myhrstad, Inger Ottestad, Magne Thoresen, Kirsten B. Holven, Stine M. Ulven

**Affiliations:** 1 Department of Nutrition, Institute of Basic Medical Sciences, University of Oslo, PO Box 1046, Blindern, 0317 Oslo, Norway; 2 Department of Nursing and Health Promotion, Faculty of Health Sciences, OsloMet – Oslo Metropolitan University, PO Box 4, St Olavs plass, 0130 Oslo, Norway; 3 Department of Biostatistics, Institute of Basic Medical Sciences, University of Oslo, PO Box 1046, Blindern, 0317 Oslo, Norway; 4 National Advisory Unit on Familial Hypercholesterolemia, Department of Endocrinology, Morbid Obesity and Preventive Medicine, Oslo University Hospital, PO Box 4950, Nydalen, 0424 Oslo, Norway

**Keywords:** EPA, DHA, Omega-3, Transcriptomics, Microarray, Triglycerides, Lipoprotein subclasses

## Abstract

**Background:**

Intake of the marine omega-3 fatty acids eicosapentaenoic acid (EPA) and docosahexaenoic acid (DHA) reduces fasting triglyceride (TG) levels and may thereby lower cardiovascular disease risk. However, there are large inter-individual differences in the TG-lowering effect of omega-3 supplementation. Genotype differences partly explain this variation, but gene-environment interactions leading to gene expression differences may also be important. In this study, we aimed to investigate baseline differences and differences in the change in peripheral blood mononuclear cell (PBMC) gene expression and lipoprotein subclass TG levels between TG responders and non-responders to omega-3 fatty acid supplementation.

**Methods:**

In a previous randomized controlled trial, healthy normotriglyceridemic subjects (*n* = 35, 71% women) received 1.6 g EPA + DHA/day for 7 weeks. In this exploratory sub-study, we defined TG responders as subjects having a TG reduction beyond the 20% day-to-day variation and non-responders as having a TG change between − 20% and + 20% after omega-3 supplementation. PBMC gene expression was measured using microarray, and lipoprotein subclasses were measured using nuclear magnetic resonance spectroscopy.

**Results:**

Eight subjects were defined as responders with a median TG reduction of 37%, and 16 subjects were defined as non-responders with a median TG change of 0%. At baseline, responders had higher TG levels in two of four high-density lipoprotein (HDL) subclasses and 909 gene transcripts (*p* ≤ 0.05) were differentially expressed compared to non-responders. During the intervention, the plasma TG reduction among responders was reflected in TG reductions in four of six different very low-density lipoprotein subclasses and three of four different HDL subclasses. Compared to non-responders, the expression of 454 transcripts was differentially altered in responders (*p* ≤ 0.05). Pathway analyses revealed that responders had altered signaling pathways related to development and immune function. In addition, two of the top 10 enriched pathways in responders compared to non-responders were related to lysophosphatidic acid signaling.

**Conclusion:**

TG responders and non-responders to omega-3 supplementation have different lipoprotein subclass and PBMC gene expression profiles at baseline and different lipoprotein subclass and PBMC gene expression responses to omega-3 supplementation. These gene expression differences may partially explain the variability in TG response observed after omega-3 supplementation.

**Graphical abstract:**

Based on free images from Servier Medical Art (Creative Commons Attribution License) and image from www.colourbox.com.
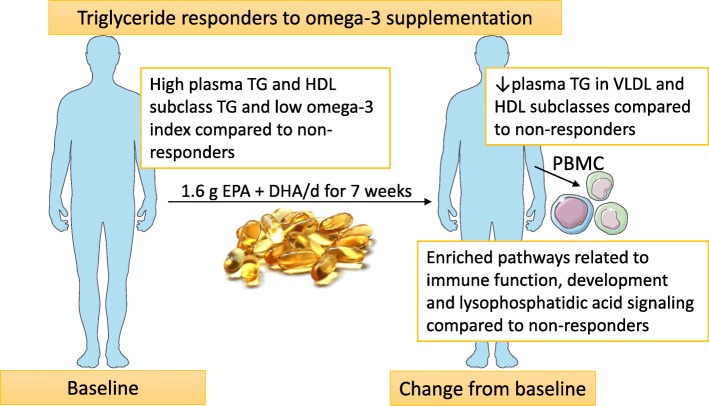

**Electronic supplementary material:**

The online version of this article (10.1186/s12263-019-0633-y) contains supplementary material, which is available to authorized users.

## Background

Intake of the marine omega-3 fatty acids eicosapentaenoic acid (EPA) and docosahexaenoic acid (DHA) may reduce the risk of cardiovascular disease (CVD), especially in high-risk populations [1]. This risk reduction is linked to the anti-inflammatory, anti-arrhythmic, blood pressure-lowering, and lipid-modifying effects of omega-3 fatty acids [2–5]. The reduction in triglyceride (TG) levels is one of the most important effects of omega-3 fatty acids, and this effect is dependent on pre-supplementation TG levels and the omega-3 fatty acid dose [2, 3]. However, there are large inter-individual differences in the TG-lowering effect of omega-3 fatty acids, with several studies showing that about 30–40% of participants do not obtain reduced TG levels following omega-3 supplementation (non-responders) [6–8]. Those who have a TG-lowering effect of omega-3 supplementation (responders) seem to have a less healthy biochemical profile, such as higher TG and glucose levels and lower HDL-C. In addition, responders have more favorable changes in total cholesterol and HDL-C in response to omega-3 supplementation than non-responders [9]. However, the distribution of TG in lipoprotein subclasses in responders and non-responders is less characterized.

Furthermore, polymorphisms in the genes apolipoprotein E (*APOE*), acetyl-CoA carboxylase α (*ACACA*), ATP citrate lyase (*ACLY*), cluster of differentiation 36 (*CD36*), retinoid X receptor α (*RXRA*), and acyl-CoA oxidase 1 (*ACOX1*), among others, have been found to affect the TG-lowering effect of omega-3 fatty acids [10–13]. It has previously been shown in a genome-wide association study (GWAS) that genotype explains only 20% of the variation in TG response to omega-3 fatty acids in the Fatty Acid Sensor (FAS) study [14]. However, in the FAS study, a more refined and improved genetic risk score (GRS) has recently showed that GRS can explain almost 50% of the TG response variance [15].

Omega-3 fatty acids mediate their effects largely at the cellular level, such as by their ability to alter gene expression. This can happen directly, by binding and activating nuclear receptors such as peroxisome proliferator-activated receptors (PPARs), or indirectly by inhibiting the nuclear translocation of transcription factors (TFs) including nuclear factor kappa B (NF-κB) and sterol regulatory element binding protein (SREBP) [16–18]. These TFs affect the transcription of genes that, among others, are involved in lipid metabolism and inflammation [19].

Peripheral blood mononuclear cells (PBMC) are cells of both the innate and adaptive immune system and are mainly composed of lymphocytes and monocytes. Because they are circulating cells, they are exposed to nutrients, metabolites, and peripheral tissues and may therefore reflect whole-body health [20, 21]. It has been shown that PBMC gene expression reflects liver and adipose tissue expression of genes involved in lipid metabolism and inflammation [22–25], and omega-3 fatty acids have been shown to alter PBMC gene expression [26–28]. Hence, PBMCs may be a good a model system for exploring the underlying mechanisms of the TG-lowering effect of omega-3 fatty acids.

In this exploratory study, we aimed to analyze the baseline differences and the difference in change in PBMC transcriptome and the TG content in lipoprotein subclasses between TG responders and non-responders to omega-3 supplementation. Since the effect of omega-3 fatty acids on lipid metabolism is partly mediated through effects on gene expression, we hypothesized that intake of omega-3 fatty acids would differentially affect PBMC gene expression in TG responders and non-responders.

## Results

### Characteristics of responders and non-responders, biochemical parameters, plasma fatty acids, and estimated omega-3 index

**Fig. 1:**
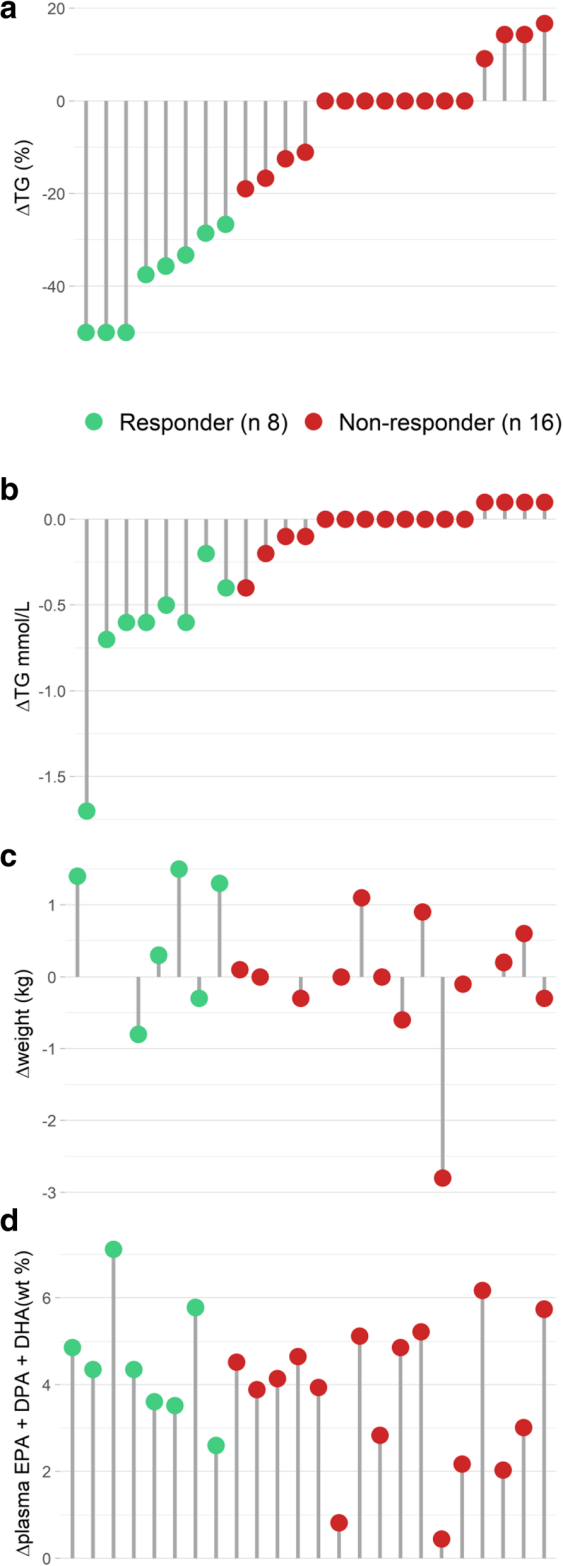
Individual changes in fasting TG, weight, and plasma levels of omega-3 fatty acids. The relative change in fasting TG levels (**a**) were used to categorize participants as responders (ΔTG ≤ − 20%, green) and non-responders (− 20% < ΔTG < + 20%, red). The participants are presented in the same order for the absolute change (mmol/L) in fasting TG levels (**b**), weight change (**c**), and the change in plasma levels of marine omega-3 fatty acids (**d**)

After 7 weeks of omega-3 supplementation, 8 participants who received omega-3 supplementation were defined as responders with a median fasting TG reduction of 37% and 16 participants were defined as non-responders with a median change in fasting TG of 0% (Fig. 1a). The median compliance estimated by capsule count was 100% in responders and 99.5% in non-responders, and the difference was not significant. At baseline, the age of the participants, the distribution of men and women, the intervention group allocation, and BMI did not differ between responders and non-responders (Table 1). Nonetheless, responders had higher baseline levels of fasting TG (*p* = 0.01) and plasma oleic acid (OA; *p* = 0.007) and lower baseline levels of plasma linoleic acid (LA; *p* = 0.009) compared to non-responders (Table 1). The baseline levels of plasma EPA, docosapentaenoic acid (DPA), and DHA did not differ between responders and non-responders. However, the baseline estimated omega-3 index was 4.6% in responders and 5.6% in non-responders and the baseline difference was significant (Table 1).

**Table 1:** Baseline characteristics, biochemical parameters, plasma fatty acids, and omega-3 index of responders and non-responders to omega-3 supplementation

	Responders (*n* = 8)							Non-responders (*n* = 16)								
	Baseline			Change 7 weeks				Baseline			Change 7 weeks				*P* **‡**	*P* **‡**
	Median/*n*	25th	75th	Median	25th	75th	*P* **†**	Median/*n*	25th	75th	Median	25th	75th	*P* **†**	Baseline	Δ 7 weeks
Gender (male/female)	3/5							5/11							1**§**	
Intervention group (FO/oxFO)	2/6							10/6							0.19**§**	
Age (years)	23.5	21.5	25.8					26.5	22.5	33					0.17	
BMI (kg/m^2^)	22.4	21.2	22.9					22.0	21.2	23.6					0.98	
Biochemical parameters																
Total-C (mM)	4.6	4.1	5.0	0.0	− 0.3	0.4	1	4.8	4.4	5.2	0.1	− 0.2	0.3	0.8	0.5	0.9
LDL-C (mM)	2.4	2.0	3.1	0.2	− 0.3	0.5	0.5	2.8	2.3	3.1	0.1	− 0.2	0.2	0.8	0.7	0.6
HDL-C (mM)	1.3	1.2	1.5	0.0	− 0.1	0.1	0.7	1.3	1.3	1.6	0.0	0.0	0.1	0.2	0.4	0.8
TG (mM)	1.5	1.4	1.7	− 0.6	− 0.6	− 0.5	0.01*	0.8	0.7	1.1	0.0	0.0	0.0	0.6	0.01*	0.0001*
Glucose (mM)	4.7	4.7	4.8	0.0	− 0.1	0.4	0.5	4.6	4.5	5.0	4.8	4.6	5.1	0.2	0.9	1
18:1 *n-9* (OA) (wt%)	24.74	21.40	26.09	− 3.86	− 5.51	− 2.25	0.01*	19.18	18.28	20.35	− 0.81	− 1.92	− 0.03	0.01*	0.007*	0.004*
18:2 *n-6* (LA) (wt%)	25.34	23.31	26.78	0.94	− 0.58	3.22	0.5	32.12	29.77	33.41	− 1.59	− 2.78	0.59	0.04*	0.009*	0.04*
18:3 *n-3* (ALA) (wt%)	0.71	0.52	0.76	− 0.23	− 0.34	− 0.04	0.01*	0.57	0.52	0.63	− 0.05	− 0.14	0.01	0.1	0.3	0.03*
20:4 *n-6* (AA) (wt%)	6.30	5.68	6.45	0.01	− 0.25	0.16	1	6.20	5.62	7.05	− 0.54	− 1.29	− 0.38	0.0004*	0.9	0.01*
20:5 *n-3* (EPA) (wt%)	0.50	0.44	0.70	1.90	1.79	2.27	0.01*	0.69	0.53	0.82	2.08	1.41	2.41	0.0005*	0.3	0.9
22:5 *n-3* (DPA) (wt%)	0.42	0.37	0.61	0.15	0.11	0.24	0.01*	0.53	0.47	0.58	0.15	0.13	0.25	0.0009*	0.6	0.9
22:6 *n-3* (DHA) (wt%)	1.70	1.46	2.01	2.35	1.57	2.67	0.01*	2.15	1.74	2.35	1.67	1.19	2.34	< 0.0001*	0.4	0.1
omega-3 index (wt%)	4.6	4.5	4.9	2.9	2.25	3.25	0.02*	5.6	4.9	6.0	2.35	1.38	3.03	0.0004*	0.03*	0.2

*25th* 25th percentile, *75th* 75th percentile, *FO* fish oil, *oxFO* oxidized fish oil, *BMI* body mass index, *C* cholesterol, *TG* triglycerides,, *n-* omega-, *OA* oleic acid, *LA* linoleic acid, *ALA* alpha-linolenic acid, *AA* arachidonic acid, *EPA* eicosapentaenoic acid, *DPA* docosapentaenoic acid, *DHA* docasahexaenoic acid, *wt%* weight%.

† Change from baseline to 7 weeks tested with paired Wilcoxon signed rank test

‡ Difference between responder and non-responder at baseline and change after 7 weeks tested with Mann-Whitney *U* test

§ Difference between responders and non-responders is tested with a Fisher’s exact test

*p < 0.05

In whole blood, there were 19% lymphocytes at baseline in both responders and non-responders, while at the 7-week visit there was 22% and 20% lymphocytes in responders and non-responders, respectively. There were 4% monocytes in both responders and non-responders at the baseline and 7-week visits. The change of lymphocytes and monocytes during the study did not differ between responders and non-responders.

During the intervention, there was no significant weight change within or between responders and non-responders (Fig. 1c). Evidently, the change in TG levels in responders and non-responders was significantly different (*p* = 0.0001) (Table 1), and as shown in Fig. 1b, the TG change ranged from − 1.7 to − 0.2 mmol/L in responders and from − 0.4 to 0.1 mmol/L in non-responders. The change in plasma EPA, DPA, and DHA did not differ between responders and non-responders (Table 1), as also illustrated by the individual changes in total plasma omega-3 levels in Fig. 1d. Nonetheless, there were significant differences between responders and non-responders in the change of several plasma fatty acids. The plasma level of OA was more reduced in responders compared to non-responders (*p* = 0.004), LA increased in responders while it decreased in non-responders (*p* = 0.04), the reduction in α-linolenic acid (ALA) was greater in responders than non-responders (*p* = 0.03), and the reduction in arachidonic acid (AA) was greater in non-responders than responders (*p* = 0.01) (Table 1).

### Lipoprotein subclasses

**Fig. 2:**
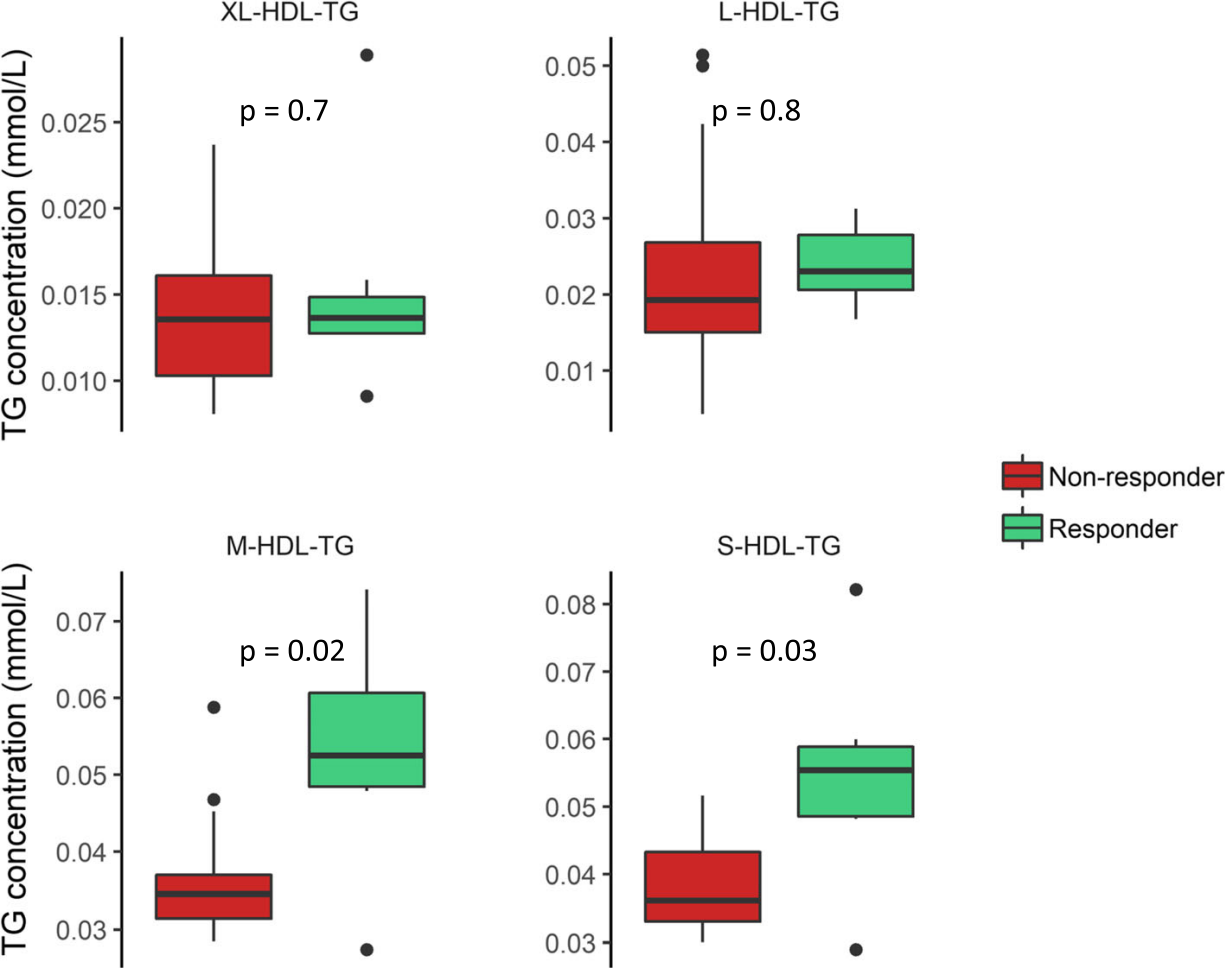
Baseline triglyceride levels in HDL subclasses. The baseline TG levels in XL-, L-, M-, and S-HDL subclasses (mmol/L) in responders (green) and non-responders (red). Differences are tested on log_2_-transformed data with an independent *t* test

**Fig. 3:**
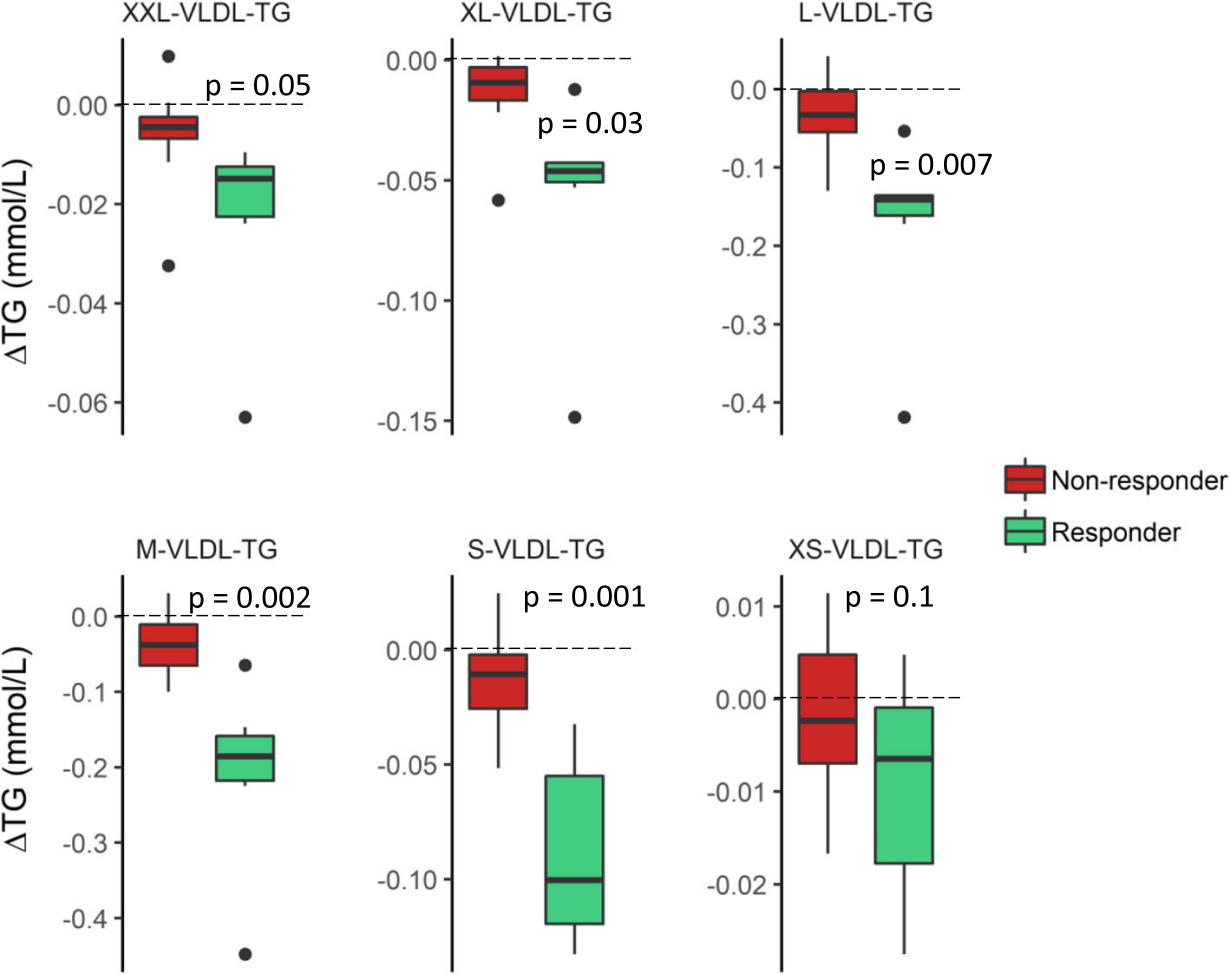
Change in triglyceride levels in VLDL subclasses. The change in TG levels in XXL-, XL-, L-, M-, S-, and XS-VLDL subclasses from baseline to end of study (Δmmol/L) in responders (green) and non-responders (red). Differences are tested on the log_2_ ratio with an independent *t* test

**Fig. 4:**
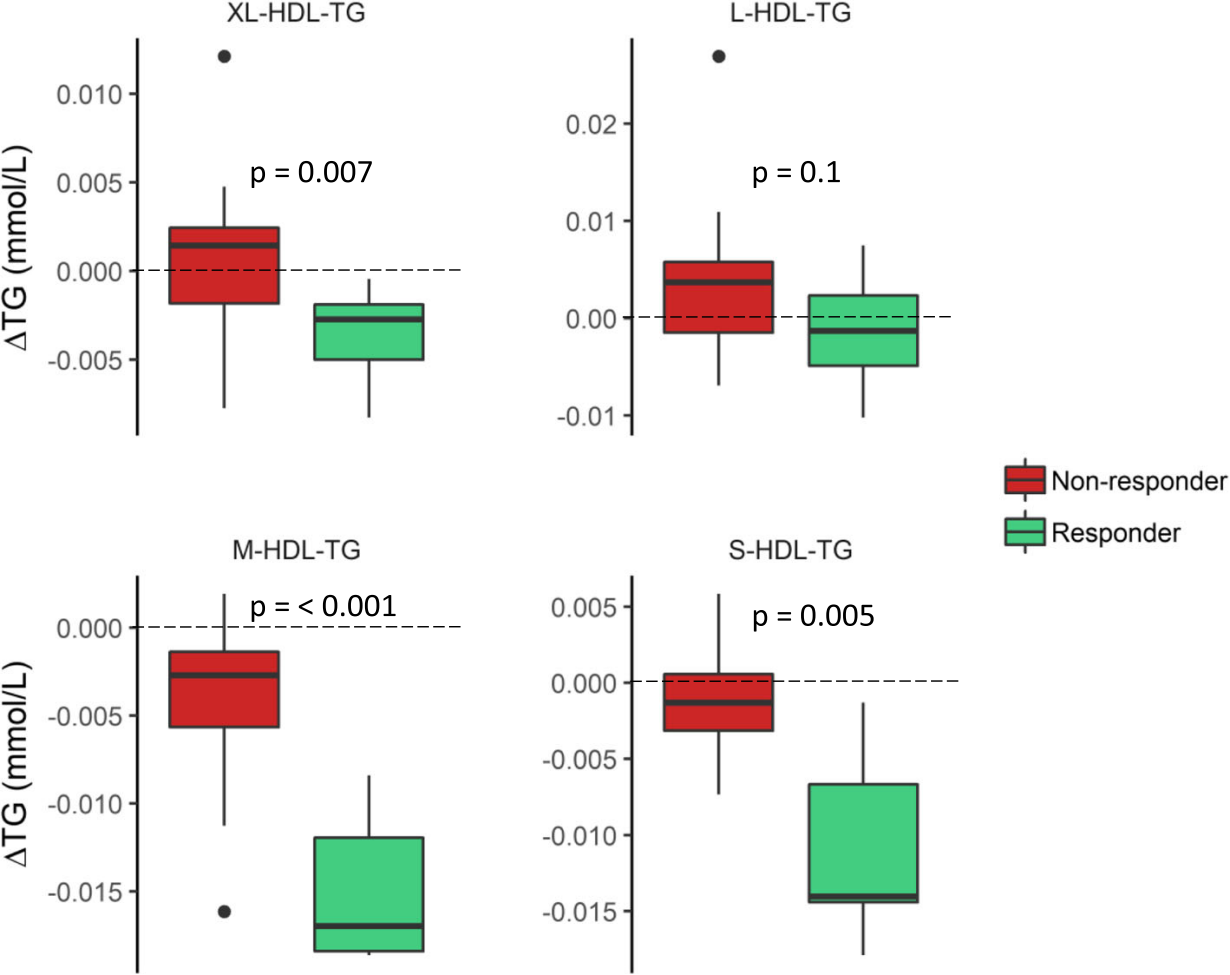
Change in triglyceride levels in HDL subclasses. The change in TG levels in XL-, L-, M-, and S-HDL subclasses from baseline to the end of study (Δmmol/L) in responders (green) and non-responders (red). Differences are tested on the log_2_ ratio with an independent *t* test

To investigate the baseline differences and the difference in change in TG levels in more detail, we analyzed TG levels in 14 different lipoprotein subclasses. At baseline, responders had higher levels of TG in the two smallest HDL subclasses, M-HDL and S-HDL (Fig. 2). However, there were no significant differences between responders and non-responders in the baseline levels of TG in any of the very low-density lipoprotein (VLDL), intermediate-density lipoprotein (IDL), or LDL subclasses (not shown). Furthermore, we found that responders had a greater reduction of TG in extra large (XL-), large (L-), medium (M-), and small (S-)VLDL subclasses (Fig. 3) as well as in the XL-, M-, and S-HDL subclasses (Fig. 4), but there were no changes in the TG levels in IDL or the LDL subclasses (not shown).

### PBMC gene expression

We analyzed the change in whole genome PBMC transcriptome at baseline and after 7 weeks of omega-3 supplementation. As previously described, all arrays fulfilled the quality criteria and were included in the analyses [27]. At baseline, 909 transcripts were differentially expressed in responders compared to non-responders (*p* ≤ 0.05). Of these transcripts, 458 transcripts had a lower expression while 451 transcripts had a higher expression in responders compared to non-responders (Additional file 1). Only one of these transcripts, the long non-coding RNA *LINC00473*, was expressed at a significantly higher level in responders after adjusting for multiple comparisons (FDR < 25%). The transcripts with the largest difference in expression at baseline (log_2_ ratio < − 0.4 and log_2_ ratio > 0.4) encoded ribosomal and cytoskeleton proteins, among others (Table 2).

**Table 2:** Genes with the highest and lowest baseline expression (log_2_ ratio > |0.4|) in responders compared to non-responders to omega-3 supplementation

Gene	Probe ID	Log ratio*	95% CI	*P* value	Biological function/process
*HBB*	ILMN_2100437	− 0.74	(− 1.47 to − 0.01)	0.05	Hemoglobin
*KRT72*	ILMN_1695812	− 0.66	(− 1.28 to − 0.05)	0.04	Cytoskeleton
*ACRBP*	ILMN_1784203	− 0.57	(− 1.08 to − 0.06)	0.03	Acrosin condensation
*UTS2*	ILMN_1732198	− 0.54	(− 1.08 to − 0.01)	0.05	Vasoconstriction
*RPL23AP7*	ILMN_2222750	− 0.52	(− 0.95 to − 0.08)	0.02	Unknown
*C21orf7*	ILMN_1699071	− 0.48	(− 0.9 to − 0.07)	0.02	Unknown
*RPL23AP7*	ILMN_1750273	− 0.43	(− 0.8 to − 0.06)	0.02	Unknown
*OST4*	ILMN_1739335	− 0.43	(− 0.77 to − 0.09)	0.01	Glycosylation of polypeptides
*RPS12*	ILMN_1679920	− 0.43	(− 0.85 to − 0.01)	0.05	Ribosomal protein
*TNNC2*	ILMN_1693428	− 0.42	(− 0.83 to − 0.01)	0.05	Cytoskeleton
*MIR130A*	ILMN_3308808	0.42	(0.05 to 0.80)	0.03	Regulation of proliferation
*GNLY*	ILMN_1708779	0.43	(0.01 to 0.85)	0.04	Inflammation
*RPS26*	ILMN_3299955	0.67	(0.01 to 1.33)	0.05	Ribosomal protein
*RPS26*	ILMN_1657950	0.68	(0.03 to 1.33)	0.04	Ribosomal protein
*RPS26*	ILMN_3291511	0.80	(0.05 to 1.55)	0.04	Ribosomal protein
*RPS26*	ILMN_1750636	1.03	(0.02 to 2.04)	0.05	Ribosomal protein

*mRNA levels in responders relative to non-responders adjusted for age and gender

After 7 weeks of omega-3 supplementation, 454 transcripts were differentially altered (*p* ≤ 0.05) in responders compared to non-responders. Of these, 246 transcripts were reduced and 208 were increased (Additional file 2). However, after correction for multiple testing, there were no differences between responders and non-responders in change in mRNA expression from baseline to 7 weeks (FDR < 25%). Among the significantly altered transcripts, those that had the greatest reduction (log_2_ ratio < − 0.25) and the highest increase (log_2_ ratio > 0.25) in responders compared to non-responders encode proteins involved in regulation of gene expression, apoptosis, and immune function (Table 3).

**Table 3:** Genes with the most altered expression (log_2_ ratio > |0.25|) in responders compared to non-responders to omega-3 supplementation

Gene	Probe ID	Difference in log ratio*	95% CI	*P* value	Biological function/process
*LINC01000*	ILMN_3234967	− 0.47	(− 0.76 to − 0.19)	0.003	Unknown
*TGFBR3*	ILMN_1784287	− 0.40	(− 0.71 to − 0.09)	0.01	Growth factor and cytokine signaling
*CCDC88C*	ILMN_3248352	− 0.37	(− 0.74 to 0.0)	0.05	Wnt signaling
*SDF4*	ILMN_2378257	− 0.33	(− 0.66 to − 0.01)	0.05	Ca^2+^ binding
*PKN1*	ILMN_2367710	− 0.31	(− 0.6 to − 0.02)	0.04	Signal transduction in apoptosis
*SNORD33*	ILMN_1682354	− 0.30	(− 0.55 to − 0.05)	0.02	Small nucleolar RNA
*NBPF10*	ILMN_2155719	− 0.29	(− 0.54 to − 0.04)	0.02	Unknown
*HNRNPL*	ILMN_2389582	− 0.28	(− 0.48 to − 0.09)	0.007	mRNA processing
*CYFIP2*	ILMN_2354478	− 0.28	(− 0.53 to − 0.03)	0.03	T cell adhesion
*PRRC2A*	ILMN_2408179	− 0.28	(− 0.55 to 0.0)	0.05	Associated with IDDM and RA development
*RFFL*	ILMN_1753819	− 0.28	(− 0.4 to − 0.15)	0.0002	Apoptosis
*FYN*	ILMN_2380801	− 0.26	(− 0.49 to − 0.03)	0.03	Proliferation
*GPKOW*	ILMN_1684197	− 0.26	(− 0.49 to − 0.02)	0.04	mRNA processing
*ZNF683*	ILMN_1678238	− 0.25	(− 0.5 to − 0.01)	0.04	T cell differentiation
*SNN*	ILMN_1788251	0.27	(0.04 to 0.51)	0.03	Regulation of growth and apoptosis
*RPL7*	ILMN_1815292	0.30	(0.01 to 0.58)	0.04	Ribosomal protein
*FTH1*	ILMN_1696911	0.32	(0.05 to 0.59)	0.02	Iron storage
*RPS3A*	ILMN_1679025	0.34	(0.03 to 0.66)	0.04	Ribosomal protein

*Change in mRNA levels from baseline to the 7-week visit in responders relative to non-responders adjusted for age and gender

*IDDM* insulin-dependent diabetes mellitus, *RA* rheumatoid arthritis

### Pathway and transcription factor analyses

To examine processes differentially altered in responders compared to non-responders after 7 weeks of omega-3 supplementation, we performed functional analyses for the 454 altered transcripts. Of the 73 enriched pathways (FDR < 25%, Additional file 3), many were related to signaling pathways involved in development and immune function. Two of the top 10 enriched pathways (Table 4) were related to lysophosphatidic acid (LPA) signaling. In addition, some pathways related to regulation of lipid metabolism, adipogenesis, blood coagulation, and thromboxane A2 signaling were enriched in responders compared to non-responders (Additional file 3).

**Table 4:** Top 10 enriched pathways in responders compared to non-responders after 7 weeks of omega-3 supplementation

Pathway maps	Ratio	*P* value	FDR	Gene transcripts
Immune response: lysophosphatidic acid signaling via NF-κB	5/53	0.0003	0.14	*ROCK2*, *IL6*, *CARD10*, *ROCK*, *TRIP6*
Inhibition of TGF-beta signaling in lung cancer	4/31	0.0003	0.14	*SERPINE1 (PAI1)*, *TAK1 (MAP3K7)*, *TGFBR3*, *SMAD6*
Immune response: IL-11 signaling pathway via MEK/ERK and PI3K/AKT cascades	5/67	0.0008	0.18	*YES1*, *HRAS*, *IL6*, *FYN*, *NCOA1*
Activation of Cortisol production in major depressive disorder	4/40	0.0009	0.18	*ASAH1*, *IL6*, *ABCB1*, *CYP11A1*
Development: ACM2 and ACM4 activation of ERK	4/43	0.001	0.18	*HRAS*, *CALD1*, *FYN*, *PLCB1*
Development: Angiotensin II/ AGTR1 signaling via RhoA and JNK	5/77	0.001	0.18	*ROCK2*, *RECK*, *SERPINE1 (PAI1)*, *PLCB1*, *ROCK*
Transcription: Androgen Receptor nuclear signaling	4/46	0.001	0.18	*HRAS*, *NCOA1*, *IL6*, *DVL3*
Immune response: M-CSF-receptor signaling pathway	5/81	0.002	0.19	*YES*, *CLB*, *HRAS*, *MSR1*, *FYN*
Chemotaxis: lysophosphatidic acid signaling via GPCRs	6/129	0.003	0.21	*HRAS*, *ROCK*, *PREX1*, *PKN1*, *TRIP6*, *PLCB*
Development: angiotensin II/ AGTR1 signaling via p38, ERK and PI3K	5/94	0.003	0.21	*RECK*, *SERPINE1 (PAI1)*, *HRAS*, *IL6*, *FYN*

The ratio indicates the number of altered transcript out of the total number of transcripts in the pathway

To further examine the mechanisms behind the differences in transcription levels between responders and non-responders, transcription factor (TF) analyses were performed. We found that genes with binding sites for 78 TFs were overrepresented in the list of genes that were significantly differentially altered in responders compared to non-responders including the TF hepatic nuclear factor 4 alpha (HNF4-α; Additional file 4).

## Discussion

In the present exploratory study, we found that intake of omega-3 fatty acids differentially altered PBMC gene expression in TG responders and non-responders. Specifically, enriched pathways in responders compared to non-responders were related to development and apoptosis, immune function, and LPA signaling. These results lend further support to the findings of Rudkowska et al. who investigated transcriptomic and metabolomics profiles of TG responders and non-responders to omega-3 supplementation. They reported that 1020 transcripts were altered within the non-responder group, and 252 transcripts were altered within the responder group with only 10 transcripts overlapping between the groups [9]. In the current study, we also report that responders had higher baseline TG in M- and S-HDL subclasses and a greater reduction in TG levels in four of six VLDL and three of four HDL subclasses than non-responders.

Among the enriched pathways in responders compared to non-responders, several were related to development signaling and immune response. In line with this, omega-3 supplementation in elderly subjects altered immune-related pathways in PBMCs [26]. This may be expected, as PBMCs are cells of the immune system, and omega-3 fatty acids alter immune responses through altering NF-κB- and PPAR-induced gene expression and by acting as precursors for the production of anti-inflammatory and pro-resolving lipid mediators [4, 29]. In addition, pathways related to immune response and apoptosis have previously been found to be altered in the group that received fish oil compared to the control oil in this study [27]. This supports that responders had altered pathways related to apoptosis and immune function compared to non-responders in this sub-study.

Pathways related to LPA signaling were enriched in responders compared to non-responders to omega-3 supplementation. LPA is a lipid containing a fatty acid that may vary in length and degree of unsaturation. LPA can be produced from membrane phospholipids; it binds and activates LPA receptors (LPAR) and different forms of LPA may differentially affect LPA signaling [30]. In addition to their role in development of the central nervous system, LPARs are expressed in lymphocytes where they affect cytokine secretion, chemotaxis, and proliferation, and LPA signaling may be involved in the development of atherosclerosis and adipocyte differentiation [30–32]. We found that responders and non-responders had different levels of various plasma fatty acids that may reflect different plasma levels of various chemical forms of LPA [30]. Similarly, Rudkowska et al. found that compared to non-responders, responders had a greater increase in unsaturated fatty acids in glycerophosphatidylcholines, which can be used as LPA precursors [9, 30].

Transcripts with binding sites for HNF4-α were overrepresented in the list of gene transcripts differentially altered in responders compared to non-responders. Long-chain PUFAs have been shown to suppress HNF4-α activity [33]. HNF4-α is a TF that induces the expression of genes involved in lipid metabolism, and a decreased HNF4-α activity has been suggested to explain decreases in serum TG levels [34]; hence, an altered activity of HNF4-α in responders may be involved in the TG-lowering effect of omega-3 fatty acids.

The baseline level and the change in plasma omega-3 fatty acid levels did not differ between responders and non-responders, indicating that omega-3 fatty acid levels in plasma are not important for the TG-lowering effect. Indeed, omega-3 fatty acids mediate many of their biological effects after being incorporated into plasma membrane phospholipids [35]. Thus, the level of omega-3 fatty acids in red blood cells (RBC), also called the omega-3 index, that indicate the level of biologically available omega-3 fatty acids may be a better measure. We report that responders had a lower estimated omega-3 index than non-responders at baseline. This may imply that responders had a lower habitual fish intake before the start of the intervention than non-responders. In line with this, only participants with a low habitual fish intake had a reduced risk of major cardiovascular events in the VITAL trial [36]. Finally, only responders had an increase in RBC DHA in the study by Rudkowska et al., indicating that the level of omega-3 fatty acids in cell membranes may be important for the TG-lowering response [9]. Coupled with the higher baseline TG levels in responders, the lower omega-3 index at baseline may explain why responders lowered their TG levels after omega-3 supplementation.

At baseline, responders had higher plasma OA levels and lower plasma LA levels than non-responders, which may reflect different dietary patterns at baseline. This may have affected the ability of omega-3 fatty acids to lower TG levels. To further understand the variation of the TG-lowering effect after omega-3 supplementation, the role of other nutrients, such as other fatty acids, in modulating this effect should be elucidated in future studies.

As expected, the TG reduction in responders was reflected by reduced TG levels in almost all VLDL subclasses. However, there was also an unexpected lowering of HDL-TG in the XL-, M-, and S-HDL subclasses. Changes in HDL-TG levels in the current study may indicate that cholesteryl ester transfer protein (CETP) could be involved, as CETP facilitates the transfer of cholesteryl esters from HDL to VLDL and LDL in the exchange for TG. Although we did not find a difference between responders and non-responders in the change in *CETP* expression, CETP activity has been shown to increase after omega-3 intake [37]. In future TG responder studies, measurements of CETP activity may provide valuable insight into possible mechanisms behind the TG-lowering effect of omega-3 fatty acids.

The classification of subjects into responders and non-responders was based on previous studies that have suggested and employed a classification of responders as those with a TG reduction, while subjects who increase or do not alter their TG levels have been classified as non-responders [7, 9]. To be more confident that the group we defined as responders actually reduced their TG levels as a response to increased omega-3 intake, we chose to only include responders with a clinically relevant TG reduction. Day-to-day variation in TG levels has been reported to be 20%; hence, a TG reduction greater than 20% in responders in this study is likely to be an effect beyond day-to-day variations [38]. Another approach would be to use the actual day-to-day TG variation in the control group in our previous study to define responders and non-responders. However, the day-to-day variation in the control group was as high as 40%, which would result in only three participants who would be defined as responders, and the statistical power of this sub-study would be low. Nonetheless, the use of actual TG variation may be useful in future studies. Moreover, the baseline TG level in this study was low, 1.5 mmol/L in responders and 0.8 mmol/L in non-responders. The TG reduction observed after omega-3 supplementation depends on the baseline TG level [3]; hence, the low baseline TG level in this study may have resulted in a lower percentage of responders compared to other studies [6, 8, 39]. Future studies investigating TG responders to omega-3 intake should include participants with high enough baseline TG levels to ensure a clinically relevant TG response.

This study is limited by the low baseline TG levels and the low number of participants. As we included fewer subjects in this sub-study compared to the main study, this study may be underpowered. In addition, no gene expression changes were significant after adjusting for multiple testing. Hence, we did not expect any detectable differences between responders and non-responders in analyses of single genes using qPCR. Therefore, the microarray results in this study are not validated by qPCR. Furthermore, an FDR limit of 25% for the enriched pathways implies that 1 in 4 of the significant results is a false positive. However, a high FDR limit was chosen to avoid losing interesting results. Hence, the results in this study needs to be interpreted with caution and validated in a study designed and powered to investigate differences between TG responders and non-responders to omega-3 fatty acid supplementation. Despite these limitations, this exploratory study found differences between responders and non-responders, and it shows that this study design may be useful for investigating the underling mechanisms of the TG-lowering effect of omega-3 fatty acids.

## Conclusions

TG responders and non-responders to omega-3 supplementation have different baseline lipoprotein subclass and PBMC gene expression profiles. Furthermore, they differentially alter their lipoprotein subclass and PBMC gene expression profiles. The differentially altered PBMC gene expression may partially explain the variability in TG response to omega-3 intake.

## Methods

### Study design and subjects

In this exploratory study, we used data from a previous double-blind randomized controlled parallel-group trial that aimed to investigate health effects of fish oils with different qualities [40]. The study was performed at Akershus University College in 2009. Healthy and non-smoking men and women aged 18–50 years with BMI < 30 kg/m^2^, TG ≤ 4 mmol/L, total cholesterol ≤ 7.5 mmol/L, C-reactive protein ≤ 10 mg/L, glucose ≤ 6 mmol/L, and blood pressure < 160/100 mmHg were included. Participants were stratified by gender and randomized in a 1:1:1 ratio to receive 1.6 g EPA + DHA per day from fish oil or oxidized fish oil or 0 g EPA + DHA per day from high-oleic sunflower oil. In total, 54 participants completed the 7-week intervention. Four weeks prior to the baseline visit and throughout the study, participants were instructed to avoid consumption of fish, omega-3 supplements, or foods enriched in omega-3 fatty acids. The first 3 weeks of the intervention was a fully controlled isoenergetic diet period, and for the remaining 4 weeks, subjects returned to their habitual diet, still avoiding consumption of omega-3 fatty acids. We have previously described subject characteristics, protocol, blinding, compliance and side effects, study products, and the fully controlled diet period in detail [40].

In the current sub-study, we combined data from the two groups that received fish oil (*n* = 35). From baseline to the 7-week visit, participants increased their intake of omega-3 fatty acids while their remaining diet was unchanged. Hence, we used data from these two visits to investigate differences in TG responders and non-responders to omega-3 fatty acids. In other studies, TG responders have been defined as all subjects with a TG reduction and non-responders as subjects who have no change or an increase in TG levels following omega-3 supplementation [7, 9]. Here, we define responders as subjects having a larger reduction in fasting TG than the 20% day-to-day variation [38] (*n* = 8) and non-responders as having a TG change between − 20 and 20% (*n* = 16). Because we wanted to compare participants with a TG reduction with those with no change in TG levels following omega-3 supplementation, participants with a higher TG increase than the 20% day-to-day variation were excluded from analyses in this study (*n* = 11).

This study was performed according to the guidelines laid down by the Declaration of Helsinki, and all procedures involving human subjects were approved by the Regional Committee for Medical Ethics (approval no. 6.2008.2215) and the Norwegian Social Science Data Services (approval no. 21924). All subjects provided written informed consent, and the study was registered at www.clinicaltrials.gov (ID no. NCT01034423).

### Blood sampling and routine laboratory analyses

Blood samples were drawn at the baseline and 7-week visits after an overnight fast (≥ 12 h). Participants were instructed to avoid alcohol consumption and strenuous physical activity the day before blood sampling. Whole blood was collected in ethylenediaminetetraacetic acid (EDTA) tubes that were kept at room temperature for maximum 48 h. Serum was obtained from silica gel tubes that were kept at room temperature for at least 30 min before centrifugation (1500***g***, 12 min), and plasma was obtained from EDTA tubes that were immediately placed on ice and centrifuged within 10 min (1500*g*, 4 °C, 10 min). Routine laboratory analyses, such as serum TG, low-density lipoprotein cholesterol (LDL-C), and high-density lipoprotein cholesterol (HDL-C), as well as white blood cell count in whole blood were performed at a clinical routine laboratory (Fürst Medical Laboratory, Oslo, Norway). Plasma fatty acids were extracted by the Bligh and Dyer method [41] as previously described [40]. The level of plasma fatty acids is expressed as the percentage of total plasma fatty acids.

### Dried blood spot omega-3 index

Whole blood from EDTA tubes were transferred to a filter paper that was dried, sealed in plastic bags, and stored at − 80 °C until analysis. The level of omega-3 fatty acids in the dried blood spots was analyzed by Vitas analytical service, Oslo, Norway. The omega-3 index was estimated from the level of whole blood EPA and DHA using an equation derived by a Norwegian population by Vitas AS (omega-3 index = whole blood EPA + DHA (%) * 0.95 + 0.35).

### Analysis of lipoprotein subclasses

The EDTA plasma concentrations of 14 different lipoprotein subclass particles and their lipid constituents, including TG levels, were measured with a commercially available NMR platform (Nightingale Health Ltd). The different lipoprotein subclasses were defined based on their average diameter: extremely large very low-density lipoprotein (VLDL) with a possible contribution of chylomicrons (> 75 nm); extra large (XL-), large (L-), small (S-), and extra small (XS-) VLDL (64.0, 53.6, 44.5, 36.8, and 31.3 nm); intermediate-density lipoprotein (IDL, 28.6 nm); L-, M-, and S-LDL (25.5, 23.0 and 18.7 nm); and XL-, L-, M-, and S-HDL (14.3, 12.1, 10.9 and 8.7 nm). Details of the NMR metabolomics platform have previously been described [42].

### Isolation of PBMC and RNA and microarray hybridization

PBMC were isolated using BD Vacutainer Cell Preparation tubes (Becton, Dickinson San Jose, CA, USA), a well-documented method for PBMC isolation with more than 90% purity. According to the manufacturer, about 80% of the isolated PBCMs are lymphocytes and 12% are monocytes. The PBMCs were isolated according to the manufacturer’s instructions and cell pellets were stored at − 80 °C. Total RNA was isolated using the Qiagen’s RNeasy Mini kit (Qiagen, Valencia, CA, USA) according to the manufacturer’s instructions. RNA quality and quantity were measured with the NanoDrop ND-1000 Spectrophotometer (Thermo Fisher Scientific, Gothenburg, Sweden) and Agilent Bioanalyzer (Agilent Technologies, Santa Clara, CA, USA). The average RNA integrity number (RIN) was 9.6. Gene expression was analyzed by hybridization to an Illumina HumanHT-12 v4 Expression BeadChip that was scanned on an Illumina HiSCan microarray scanner (Illumina Inc., San Diego, CA, USA). Illumina GenomeStudio was used to transform bead-level data to probe-level intensities, which were exported raw for bioinformatics analysis as previously reported [27].

### Analyses of microarray data

The Illumina intensities were quantile normalized, and probes without a detectable expression in at least 10% of the samples were excluded (detection *P* > 0.01). Of the 48,000 probes, 21,236 probes on the Illumina array were defined as expressed. A more detailed protocol of the microarray analyses has previously been reported [27]. We calculated the change in gene expression as the log_2_ ratio between intensities at baseline and after 7 weeks. The differences in gene expression changes between responders and non-responders were tested with multiple regression analyses adjusted for age and gender. Differentially expressed genes (*p* ≤ 0.05) were subjected to pathway analyses and transcription factor (TF) analyses using MetaCore (GeneGo, division of Thomson Reuters, St Joseph, MI, USA), and enriched pathways with a false discovery rate (FDR) < 25% were considered significant.

### Other statistical analyses

Sample size calculation for the main study was based on the expected change in plasma omega-3 levels and has previously been described [40]. Lipoprotein subclass TG levels were log_2_-transformed before baseline differences and the difference in change from baseline (log_2_ ratio) between responders and non-responders were tested with a *t* test. Differences in categorical data were tested with Fisher’s exact test. All other data are presented as median and 25th–75th percentiles. The differences from baseline to the 7-week visit within a group were tested with a paired Wilcoxon signed rank test and the differences between responders and non-responders at baseline and the differences in change from baseline to the 7-week visit were tested with a Mann-Whitney *U* test. All statistical analyses were performed in R [43].

## Additional files

Additional file 1: List of genes with a significantly (*p* ≤ 0.05) different baseline expression in responders compared to non-responders adjusted for age and gender. (XLSX 671 kb)

Additional file 2: List of significantly (*p* ≤ 0.05) differentially altered gene transcripts in responders compared to non-responders adjusted for age and gender. (XLSX 622 kb)

Additional file 3: Enriched pathways (FDR < 25%) among the significantly altered transcripts in responders compared to non-responders. (XLSX 14 kb)

Additional file 4: Results from the transcription factor analyses. (XLSX 16 kb)

## Abbreviations

AA: Arachidonic acid
ACACA: Acetyl-CoA carboxylase α
ACLY: ATP citrate lyase
ACOX1: Acyl-CoA oxidase 1
ALA: α-Linolenic acid
APOE: Apolipoprotein E
BMI: Body mass index
C: Cholesterol
CD36: Cluster of differentiation 36
CETP: Cholesteryl ester transfer protein
CVD: Cardiovascular disease
DGAT2: Diacylglycerol O-acyltransferase 2
DHA: Docosahexaenoic acid
EDTA: Ethylenediaminetetraacetic acid
EPA: Eicosapentaenoic acid
FDR: False discovery rate
FO: Fish oil
GWAS: Genome-wide association study
HDL: High-density lipoprotein
HNF4A: Hepatic nuclear factor 4α
IDL: Intermediate-density lipoprotein
L-: Large
LA: Linoleic acid
LDL: Low-density lipoprotein
LPA: Lysophosphatididic acid
LPAR: Lysophosphatididic acid receptor
M-: Medium
NF-κB: Nuclear factor kappa B
NMR: Nuclear magnetic resonance
OA: Oleic acid
oxFO: Oxidized fish oil
PBMC: Peripheral blood mononuclear cells
PPAR: Peroxisome proliferator-activated receptors
RBC: Red blood cell
RXRA: Retinoid X receptor α
S-: Small
SREBP: Sterol regulating element binding protein
TF: Transcription factor
TG: Triglycerides
TGF-beta: Transforming growth factor β
VLDL: Very low-density lipoprotein
XL-: Extra large
XS-: Extra small
XXL-: Extremely large
